# Supplementary material for: Preclinical validation of a novel metastasis‐inhibiting Tie1 function‐blocking antibody
Source: EMBO Mol Med. 2020 Apr 17;12(6):e11164. doi: 10.15252/emmm.201911164 (PMC7278563; doi:10.15252/emmm.201911164)
Supplement: Supplementary file 1 — Expanded View Figures PDF [file EMMM-12-e11164-s001.pdf]

Expanded View Figures

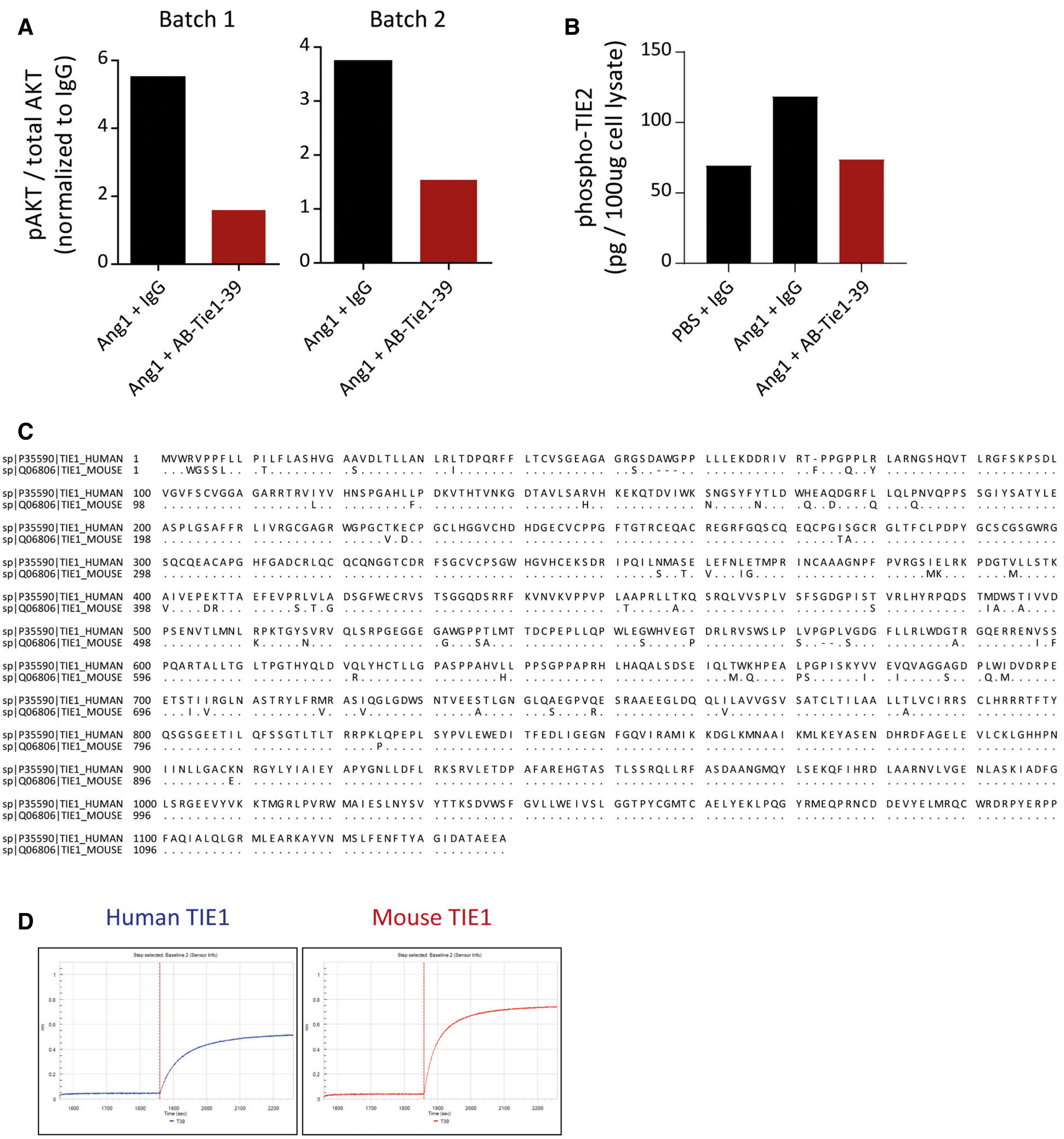

Figure EV1. Screening and characterization of Tie1-binding antibodies.

A AB-Tie1-39 was produced in two independent batches that were independently evaluated for their efficacy to inhibit Tie2 activation (n = 1 experiment for two batches of antibody production).

B ELISA-based quantitation of phospho-TIE2 amounts in HUVEC lysate stimulated with Ang1 and treated either with IgG or with AB-Tie1-39 (mean of three independent *in vitro* replicates is shown).

C Sequence comparison between human and mouse Tie1 shows 92.62% homology. A dot in the TIE1_MOUSE sequence implies a conserved amino acid residue.

D Biacore binding curves demonstrating the high-affinity binding of AB-Tie1-39 to both human and mouse Tie1.

© 2020 The Authors

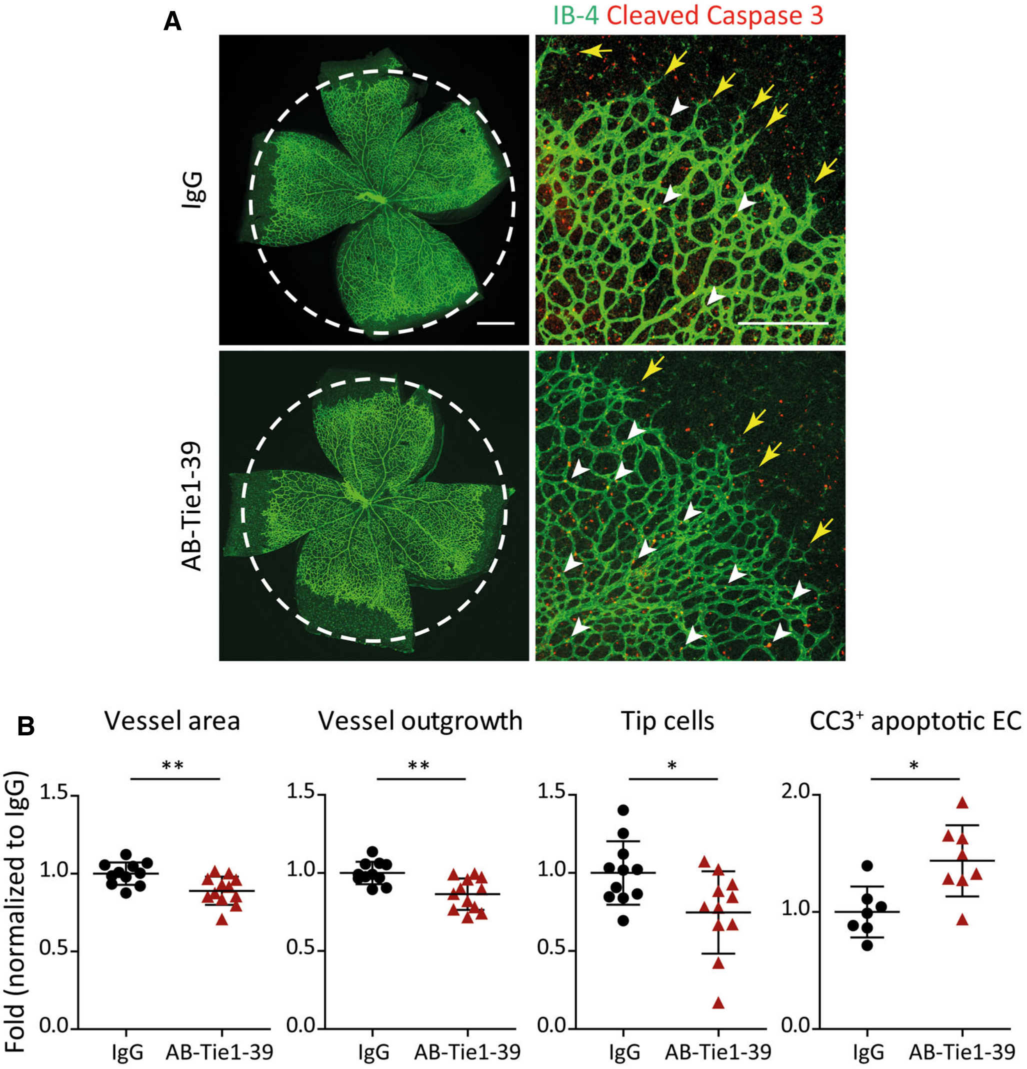

Figure EV2. Administration of AB-Tie1-39 suppresses retinal angiogenesis.

A Representative whole-mount images of retinas from mice treated with either IgG or AB-Tie1-39. Yellow arrows highlight angiogenic tip cells, and white arrowheads indicate cleaved caspase 3 (CC3)-positive EC (labeled in red). The scale bars represent 500 μm (left panel) and 150 μm (right panel).

B Dot plots show quantitation of vessel area (mean ± SD, n_{IgG} = 11, $n_{AB\text{-}Tie1\text{-}39}$ = 12 retinas) and outgrowth (mean ± SD, n_{IgG} = 11, $n_{AB\text{-}Tie1\text{-}39}$ = 12 retinas), the number of tip cells (mean ± SD, n_{IgG} = 11, $n_{AB\text{-}Tie1\text{-}39}$ = 11 retinas), and CC3-positive apoptotic EC (mean ± SD, n_{IgG} = 7, $n_{AB\text{-}Tie1\text{-}39}$ = 8 retinas). *$P < 0.05$; **$P < 0.01$ (two-tailed Mann–Whitney *U*-test).

© 2020 The Authors

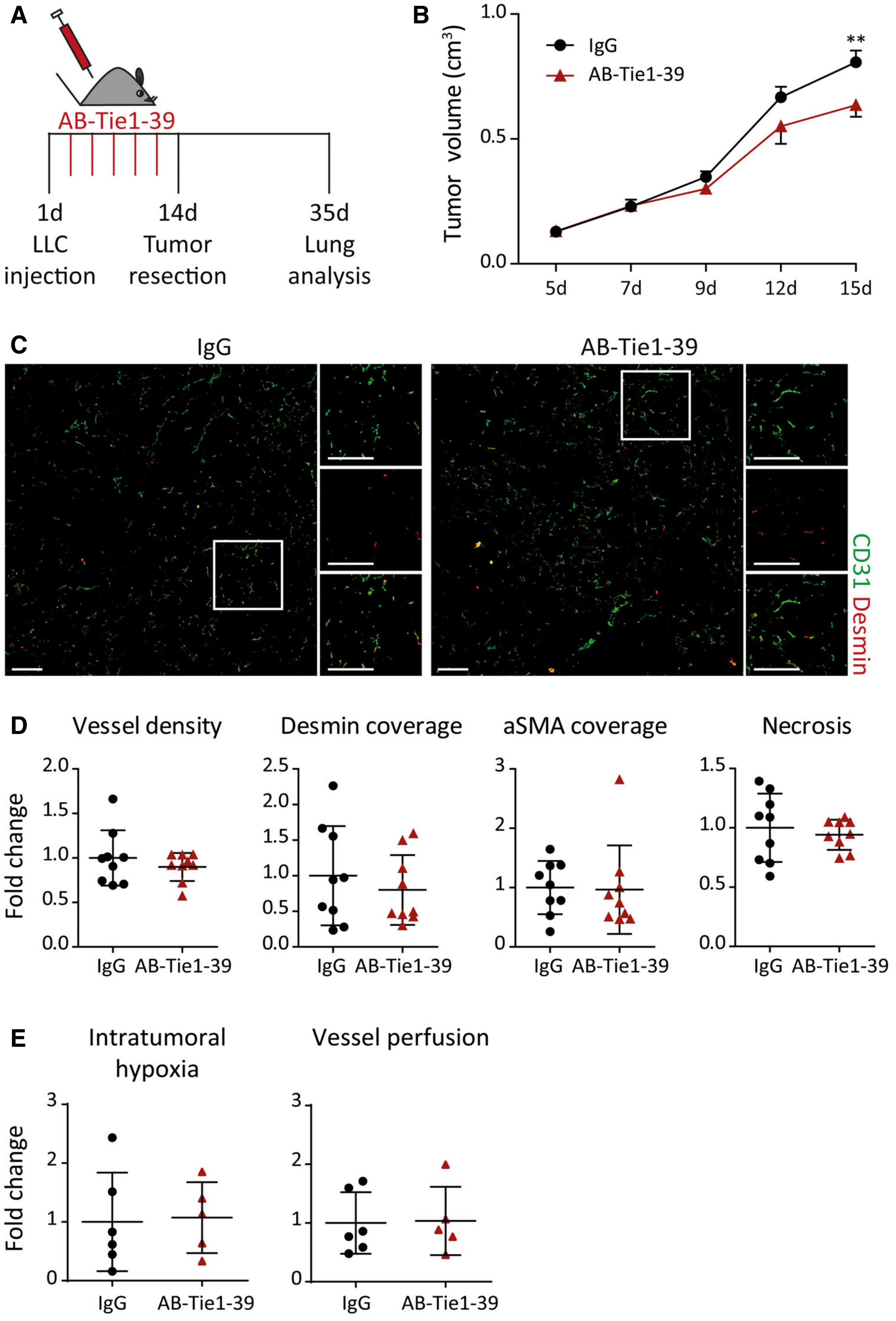

Figure EV3. Neoadjuvant treatment with AB-Tie1-39 slows the growth of LLC tumors with no overt vascular defects.

A Experimental outline of spontaneous metastatic LLC cancer model treated with IgG or AB-Tie1-39 in a presurgical neoadjuvant setting.

B Tumor growth curves displaying delayed primary tumor growth when treated with AB-Tie1-39 antibody as compared to IgG treatment (mean ± SEM, $n = 9$ mice). $^{**}P < 0.01$ (two-way ANOVA test).

C Representative immunofluorescence images of tumor sections stained with CD31 (EC-specific marker) and Desmin (mural cell-specific marker). Scale bars = 200 μm.

D Dot plots showing quantitation of intratumoral vessel density, mural cell coverage using Desmin and aSMA co-staining, and tumor necrosis (mean ± SD, $n = 9$ mice).

E Dot plots showing quantitation of intratumoral hypoxia and vessel perfusion analysis (mean ± SD, $n_{IgG} = 6$, $n_{AB\text{-}Tie1\text{-}39} = 5$ mice).

Data information: (D, E) All comparisons were rendered non-significant according to two-tailed Mann–Whitney *U*-test.

 © 2020 The Authors

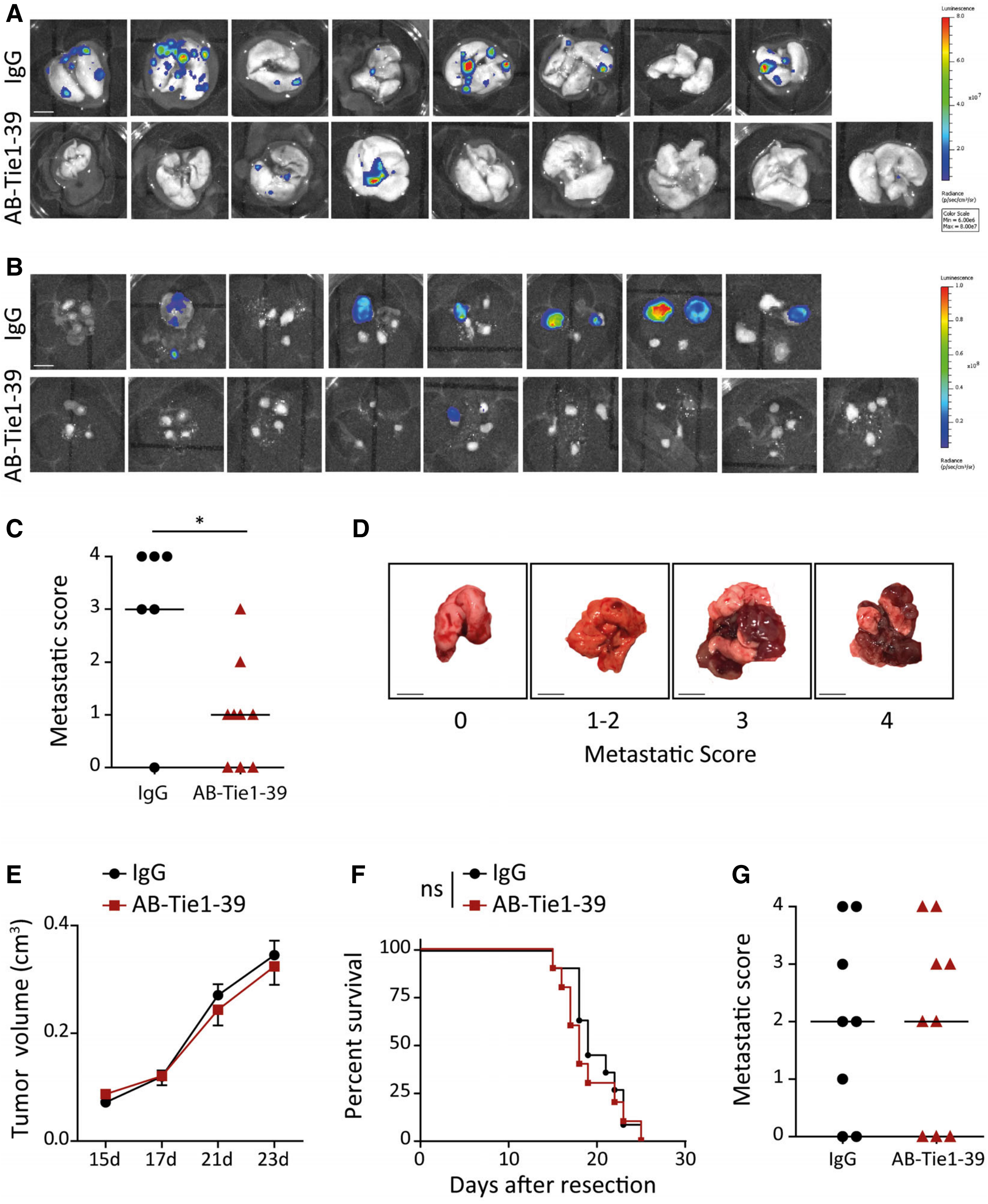

Figure EV4. Neoadjuvant but not adjuvant treatment with AB-Tie1-39 inhibits metastasis.

A, B In the 4T1 tumor model, mice were sacrificed 2 weeks after primary tumor resection and *ex vivo* bioluminescence imaging was performed on the isolated metastatic organs. Shown are the BLI images for lung (A) and lymph nodes (B). Scale bars = 5 mm.

C In the LLC metastasis model, mice were sacrificed following a neoadjuvant treatment regimen with IgG or AB-Tie1-39 3 weeks after primary tumor resection. The lungs were analyzed and scored for metastatic burden as shown in the dot plot (mean ± SD, n_{IgG} = 6, $n_{AB\text{-}Tie1\text{-}39}$ = 9 mice). *$P < 0.05$ (two-tailed Mann–Whitney *U*-test).

D Representative lung images associated with the different metastatic scores. Scale bars = 5 mm.

E Growth curve illustrating progression of 4T1 tumors when treated with IgG or AB-Tie1-39 in a perioperative therapeutic regimen (mean ± SEM, n_{IgG} = 11, $n_{AB\text{-}Tie1\text{-}39}$ = 10 mice). The comparison was rendered non-significant according to *two-way* ANOVA *test*.

F Kaplan–Meier plot showing percent survival of mice after primary tumor resection and postsurgical adjuvant treatment with IgG or AB-Tie1-39 in the 4T1 model (n_{IgG} = 11, $n_{AB\text{-}Tie1\text{-}39}$ = 10 mice). The comparison was rendered non-significant (ns) according to *log-rank* (Mantel–Cox) test.

G Dot plot representing the lung metastatic score for mice treated with either IgG or AB-Tie1-39 (n_{IgG} = 8, $n_{AB\text{-}Tie1\text{-}39}$ = 9 mice). The comparison was rendered non-significant according to two-tailed Mann–Whitney *U*-test.

Data information: (C, F–G) Mice with primary tumor regrowth after tumor resection were excluded from the analysis.

© 2020 The Authors

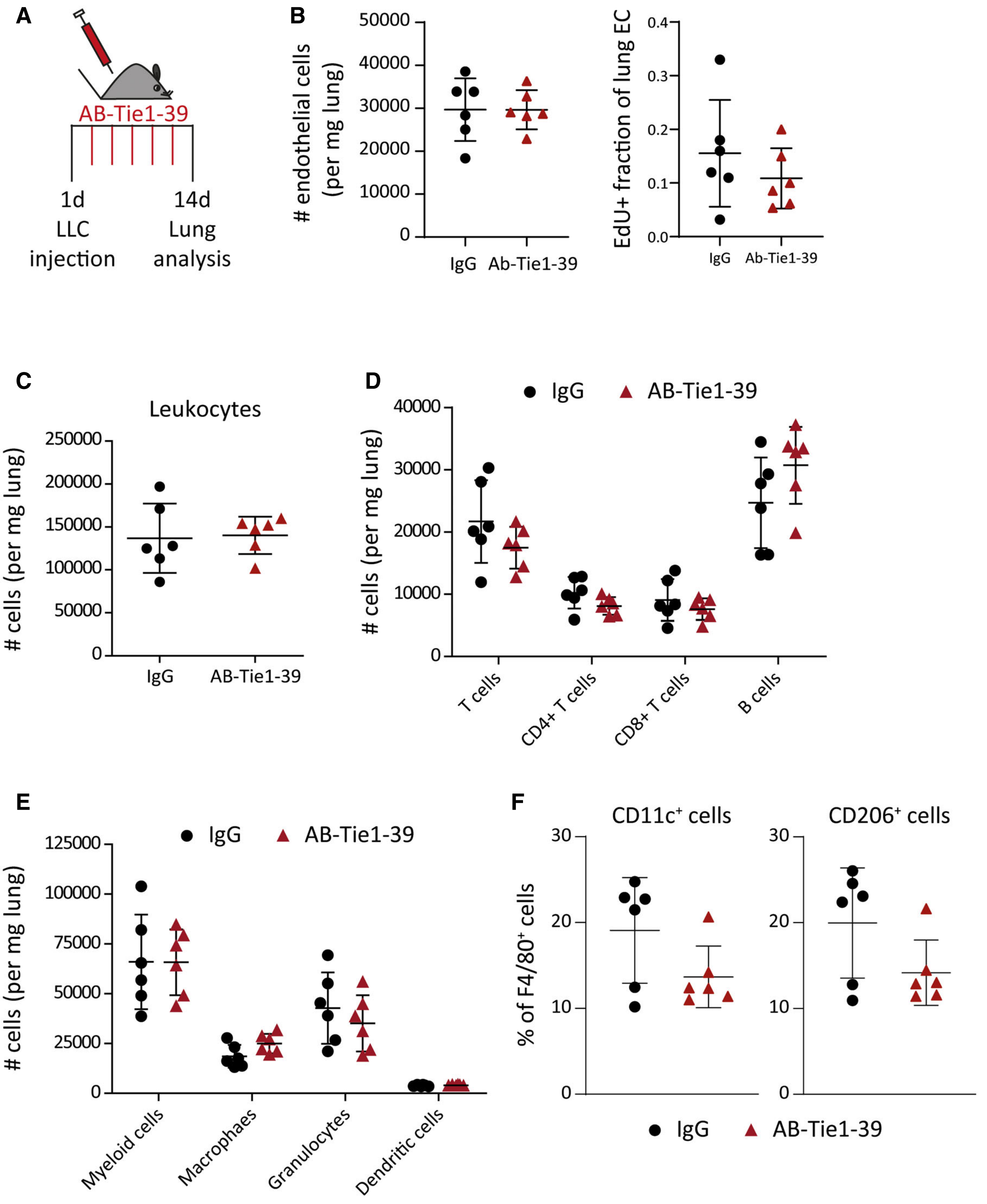

Figure EV5. Neoadjuvant treatment with AB-Tie1-39 did not alter the immune landscape in the lungs.

A Mice bearing LLC primary tumors were treated with IgG or AB-Tie1-39 in a neoadjuvant manner. Detailed FACS-based analyses were performed on the lung tissues.
B Dot plot indicating total number and proliferating fraction of lung EC (mean ± SD, *n* = 6 mice).
C Dot plot indicating the number of leukocytes (mean ± SD, *n* = 6 mice).
D The graph shows the number of different lymphoid cell populations (mean ± SD, *n* = 6 mice).
E The graph represents the number of different myeloid cell populations (mean ± SD, n = 6 mice).
F The graphs illustrate percentages of M1 ($CD11c^+$) and M2 ($CD206^+$) macrophages (mean ± SD, *n* = 6 mice).

Data information: (B–F) All comparisons were rendered non-significant according to two-tailed Mann–Whitney *U*-test.

 © 2020 The Authors
