# Supplementary figures and images for: Preclinical validation of a novel metastasis‐inhibiting Tie1 function‐blocking antibody
Source: EMBO Mol Med. 2020 Apr 17;12(6):e11164. doi: 10.15252/emmm.201911164 (PMC7278563; doi:10.15252/emmm.201911164)

## Source Data - Figure 3D

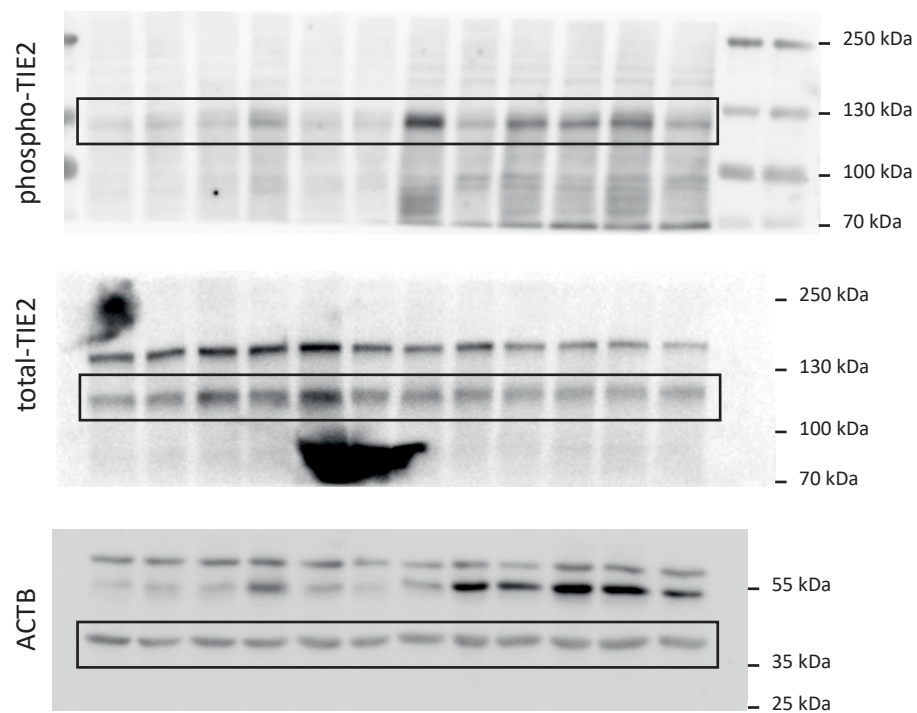

Boxed area of each blot is shown in Figure 3D.

Supplement: Supplementary file 3 — Source Data for Figure 3 [file EMMM-12-e11164-s002.pdf]
